# Autonomous precision resuscitation during ground and air transport of an animal hemorrhagic shock model

**DOI:** 10.1186/s40635-024-00628-5

**Published:** 2024-05-24

**Authors:** Michael R. Pinsky, Hernando Gomez, Francis X. Guyette, Leonard Weiss, Artur Dubrawski, Jim Leonard, Robert MacLachlan, Lisa Gordon, Theodore Lagattuta, David Salcido, Ronald Poropatich

**Affiliations:** 1 Department of Critical Care Medicine, University of Pittsburgh School of Medicine, 638 Scaife Hall, 3550 Terrace Street, Pittsburgh, PA 15261 USA; 2 Center for Military Medicine Research, University of Pittsburgh, Pittsburgh, PA USA; 3 Department of Emergency Medicine, University of Pittsburgh School of Medicine, Pittsburgh, PA USA; 4 Auton Lab, School of Computer Science, Carnegie-Mellon University, Pittsburgh, PA USA; 5 Division of Pulmonary, Allergy, Critical Care and Sleep Medicine, University of Pittsburgh School of Medicine, Pittsburgh, PA USA

**Keywords:** Animal model, Closed-loop, Hemorrhagic shock, Resuscitation, Transport

## Abstract

We tested the ability of a physiologically driven minimally invasive closed-loop algorithm, called Resuscitation based on Functional Hemodynamic Monitoring (ReFit), to stabilize for up to 3 h a porcine model of noncompressible hemorrhage induced by severe liver injury and do so during both ground and air transport. Twelve animals were resuscitated using ReFit to drive fluid and vasopressor infusion to a mean arterial pressure (MAP) > 60 mmHg and heart rate < 110 min^−1^ 30 min after MAP < 40 mmHg following liver injury. ReFit was initially validated in 8 animals in the laboratory, then in 4 animals during air (23nm and 35nm) and ground (9 mi) to air (9.5nm and 83m) transport returning to the laboratory. The ReFit algorithm kept all animals stable for ~ 3 h. Thus, ReFit algorithm can diagnose and treat ongoing hemorrhagic shock independent to the site of care or during transport. These results have implications for treatment of critically ill patients in remote, austere and contested environments and during transport to a higher level of care.

**Supplementary Information:**

The online version contains supplementary material available at 10.1186/s40635-024-00628-5.

Resuscitation of severe hemorrhagic shock requires rapid and appropriate time-dependent care to decrease morbidity and mortality. Most severe traumatic hemorrhage events occur in the field remote from acute care services. Once first responder services arrive their diagnostics are limited to gross examination and measures of vital signs. Similarly, first responders are rarely able to perform definitive resuscitation but may temporize the patient with external hemorrhage control and infusions of fluid and blood, if available. These efforts aim to stabilize the patient long enough to transport them to locations where definitive surgical interventions can be obtained, while preserving end organ function [1]. These scenarios are further complicated by delay and austerity which occur during remote site trauma, civilian mass casualty, war and natural disasters. If a fully autonomous (i.e., closed loop) diagnosis and resuscitation algorithm was linked to routinely available non-invasive or minimally invasive hemodynamic monitoring, unstable trauma patients could be identified and temporized for prolonged time intervals allowing safe transfer to higher levels of care with less deterioration [2]. In August 2023 the Economist reported that the use of supply delivery drones to evacuate the wounded to field hospitals up to 45 km away from the front line [3]. This was the first-time transport of the wounded was done on an unmanned aerial vehicle without human support.

We previously proposed a physiologically based closed loop control (PCLC) resuscitation algorithm to diagnose and treat hemorrhagic shock based on validated dynamic physiologic measures easily acquired from existing invasive or minimally invasive hemodynamic monitoring. This PCLC structure is referred to as Resuscitation based on Functional Hemodynamic Monitoring (ReFit) [4]. We subsequently demonstrated that in a porcine severe hemorrhagic shock model created by a fixed rate of blood withdrawal to a threshold minimal mean arterial pressure (MAP), modeling severe compressible hemorrhage (e.g., traumatic limb laceration/amputation) our ReFit algorithm could identify cardiovascular insufficiency, guide proportional resuscitation and have clear stopping rules to prevent over-resuscitation [5, 6]. Furthermore, time to initial resuscitation using the ReFit algorithm was similar to that of a senior critical care physician doing open resuscitation. In the present work, using a porcine model of severe uncontrolled hemorrhagic shock induced by a liver laceration with a one-hour 50% mortality if untreated, we asked the question can this ReFit algorithm effectively run using only minimally invasive arterial pressure monitoring and without the human intervention and both can rescue all swine and sustain hemodynamic stability for up to 3 h after injury even during prolonged ground and air transport. We chose 3 h because that is enough time usually needed to transport such victims to an advanced aid station for definitive care. If shown to be effective, this approach has the potential to support patients in the setting of trauma occurring in remote or contested areas from which transport times are prolonged and to expand the capability of supporting multiple patients when human and expert resources are lacking as is often seen in war [3] or could be seen in a civilian mass casualty scenario, as is often needed to place such patients in advanced aid stations. This would also extend the “Golden Hour” of time needed to start definitive surgical care post-trauma to hours.

## Methods

### Animal preparation and surgical procedures

All experiments were performed in accordance with the National Institutes of Health Animal Research: Reporting of In Vivo Experiments guidelines [7] and under protocols approved by the Institutional Animal Care and Use Committee of the University of Pittsburgh (IACUC protocol No. IS00015990) for ethical validation of need, minimizing of pain and discomfort, selection of species, strain, number and sex of the animals, and Animal Care and Use Review Office (ACURO), a component of the USAMRDC Office of Research Protections (https://mrdc.health.mil/index.cfm/collaborate/research_protections/acuro). The surgical preparation and experimental design have been previously described [6]. Briefly, 15 female pigs (30.8 ± 0.7 kg body weight) were anesthetized with inhaled isoflurane at ~ 2% and ventilated with a tidal volume of 8 ml/kg and 5 cmH_2_0 positive end-expiratory pressure and FiO_2_ of 0.4.. They were then instrumented with a femoral arterial catheter to measure arterial pressure and sample blood and internal jugular catheter to withdraw blood during exchange transfusions and to administer fluids and drugs as described below during resuscitation. A pulmonary artery catheter (Edwards Lifesciences) was placed through internal jugular port to continuously monitor cardiac output and mixed venous O_2_ saturation (SvO_2_). The arterial pressure signal was transduced through a LiDCOplus® (LiDCO, London) monitor and reported beat-to-beat stroke volume, stroke volume variation (SVV), pulse pressure variation (PPV) and PPV to SVV ratio, called dynamic arterial elastance (Ea_dyn_). A pulse oximeter was placed on the ear and pulse oximeter oxygen saturation and the plethysmographic output recorded (Co-pilot, Nonin Medical). An exchange transfusion of 500 ml blood was done in 50-ml aliquots with lactated Ringer’s solution and the collected blood saved in blood bank storage bags at room temperature for subsequent infusion. A midline laparotomy was performed but held loosely closed with four stay sutures after insertion of a Foley catheter in the urinary bladder. After instrumentation, total intravenous anesthesia using continuous infusions of ketamine and fentanyl was instituted, the inhaled anesthetic was weaned. Ketamine and fentanyl were given through computer-controlled infusion pumps (Neurowave, Cleveland), with their rates defined during the baseline interval. Animals were monitored for signs of inadequate sedation and analgesia and infusion of ketamine and fentanyl adjusted accordingly to maintain a stable level of anesthesia throughout the remainder of the experiment. In practice, no changes in ketamine or fentanyl were necessary after baseline, even during ground and air transport. The animals were switched to an automated Hamilton T1 ventilator at the same ventilator settings for the remainder of the experiment. A 30-min period was allowed for stabilization after surgical preparation and the mean hemodynamic values during the final 5 min were used to define each animal’s hemodynamic baseline.

### Uncontrolled hemorrhagic shock protocol

After 30-min baseline interval the abdomen was reopened and two 2X2 cm through and through lacerations of the right lobe of the liver were performed, as previously described [8]. The animals were observed until the mean arterial pressure (MAP) decreased to < 40 mmHg over > 80% of a 5-min moving window. At which time 12 of the 15 animals had the surface of the liver was packed with surgical pads and the abdomen closed in two layers. In three animals no packing, skin closure or resuscitation was done post-laceration to define the natural history of this severe insult. In the remaining 12 animals 30 min after reaching the initial hypotension threshold of 40 mmHg the ReFit algorithm was started and continued for ~ 3 h post-insult. Serum lactate levels and blood gases were measured immediately prior to liver laceration and at the start of resuscitation and then periodically thereafter.

### The ReFit algorithm

We created a physiologically based controlled diagnosis and resuscitation system based on continuous measures of MAP, heart rate (HR), arterial PPV, SVV and Ea_dyn_, to drive four computer-controlled infusion pumps delivering whole blood, CaCl_2_, lactated Ringer’s solution and norepinephrine (Neurowave Medical, Cleveland, OH). The previously reported [6] ReFit algorithms are also described in Additional file 1. The ReFit algorithm used MAP and HR thresholds to define need for resuscitation from circulatory shock for several years and are endorsed by most major critical care societies and the Surviving Sepsis Resuscitation guidelines. The diagnosis and treatment process was a 15-min cycle in three 5-min phases. Specific bolus of lactated Ringer’s solution or change in vasopressor infusion rate were given during the first 5 min, waiting 5 min for equilibration and the last 5 min reassessing MAP and HR to define the next interval’s treatment. The ReFit protocol used MAP < 60 mmHg and HR > 110 min^−1^ as the thresholds for initiating resuscitation and their absence as stopping rules. However, the ReFit algorithm allows the operator to set the target value of MAP to any level. Consistent with current recommendations of field resuscitation of hemorrhagic trauma protocols [8], at the start of resuscitation 250 ml whole blood (equal to one unit of blood for a 70 kg human) was infused over 5 min followed by 1 gm CaCl_2_ infused over 5 min. To match the usual field care of a trauma patient, a second 250 ml whole blood was then infused if MAP and HR stopping rules were not met. From this point on, if the subject remained hypotensive and/or tachycardic, graded boluses of lactated Ringer’s solution or norepinephrine were initiated based on the physiologic responses of each individual animal. Specifically, lactated Ringer’s solution boluses were given based on PPV threshold sub-routine: PPV < 10% received no infusion; while fluid infusion was given if PPV ≥ 10% proportional to the degree of volume responsiveness by the following scale: PPV ≥ 10% to < 30% 5 ml·kg^−1^; PPV ≥ 30 to < 50% 8 ml·kg^−1^; and PPV ≥ 50% 10 ml·kg^−1^ lactate Ringer’s solution were delivered. The choice of these levels and PPV ranges were chosen arbitrarily based on 5 ml·kg^−1^ being the minimal fluid challenge usually given and 10 ml·kg^−1^ being the maximal amount of fluid that can be safely given over 5 min, with 8 ml·kg^−1^ being in the middle. PPV is the ratio of the difference between maximal and minimal diastolic to systolic arterial pressure (PPmax-PPmin)) to mean diastolic to systolic pulse pressure (PPmean) over a 20-s moving window ([PPmac-PPmin]/PPmean). If MAP was < 60 mmHg after the first lactated Ringer’s infusion, a fourth pump would deliver continuous norepinephrine infusions started at 0.03 μg·kg^−1^·min^−1^. Subsequent changes in norepinephrine were in 0.01 microg/kg/min steps up to a maximum infusion rate of 0.3 μg·kg^−1^·min^−1^. If MAP remained > 60 mmHg for 15-min cycle without need for further fluid boluses and Ea_dyn_ was > 1, then norepinephrine was decreased. The Refit algorithm was continued for ~ 3 h after starting resuscitation. The main decision tree logic and the fluids and norepinephrine sub-routines are described in Additional file 1.

We defined time to initial stabilization as the start of the first 15-min cycle after an initial lactated Ringer’s solution was given in which no treatments were given or changed. However, monitoring and subsequent algorithm-based resuscitation, if needed, were continued for ~ 3 h after liver injury to define longer-term sufficiency (i.e., sustaining MAP and HR targets).

### Experimental groups

Eight animals were studied in the laboratory to validate and calibrate the ReFit algorithm in this uncontrolled hemorrhagic shock model. Three additional animals received no treatment to characterize the natural history of major liver laceration. The final 4 animals were studied both in the laboratory and during transport and return to the laboratory. Transport was started once the animals had received the initial 250 ml of whole blood and CaCl_2_ to model the initial treatment of severe hemorrhage in the field prior to transport [9]. The animals and the infusion pump manifold, ruggedized computer and pressure monitoring system were first moved from the surgical table to a stretcher. In practice this took about 5 min. The ReFit algorithm ran continuously independent of the location of the animal, whether during transfer to the stretcher, moving by stretcher to the vehicle, during air or ground transport, or after returning to the laboratory. The first two animals modeled an interfacility air patient transfer by STAT MedEvac clinicians. The animals were transported to the rooftop helipad and loaded onto an Airbus H135 helicopter. Two medical safety officers were on board only to monitor vital signs and administer anesthetics if signs of inadequate anesthetic plane (rigors) were seen, but they did not monitor or alter the ReFit protocol. The first flight lasted 24 min covering 39.2 nautical miles (nm) and the second flight was 36 min covering 66.4 nm prior to returning to the helipad and transferring the animals back to the laboratory. To model a rendezvous at a remote landing zone, the third and fourth animals were transported to the hospital loading bay, placed in a STAT MedEvac ground ambulance and driven to a remote airport (Allegheny County airport) where a waiting STAT MedEvac helicopter crew collected the animal, placed it in the Airbus H135 helicopter and returned them to the hospital helipad and finally to the laboratory. It took 30 min to drive the 9 miles to the remote airport. The first ground to air transport animal took 12 min air transport covering 9.1 nm, and the second ground to air transport took 35 min covering 62.3 nm to simulate a longer evacuation distance. Examples of the infusion pump manifold, graphic display and ReFit routine during transport and pictures of the processes of transporting the animals as well as the flight paths of the four animals are shown in Additional file 1. The ability to remotely monitor the ReFit protocol during transport from our laboratory system, and to change infusion rates of the intravenous anesthesia pumps was also tested in the last two transported animals to validate that remote monitorng and control were possible, but no changes were made to the ReFit controlled infusion pumps.

## Results

All 12 animals treated with the ReFit protocol survived and were hemodynamically stable up until the 3-h post-liver laceration stopping point (Table 1). The 8 laboratory-only animals reached initial stability at 48.7 ± 18.4 min and the four transported animals reached stability in 42.4 ± 30.2 min. These times to reach the initial hemodynamic stability level were similar to those reported in our prior study using a controlled hemorrhage model and similar to the times to initial resuscitation for a senior critical care physician openly resuscitating such animals [6]. Conversely, two of the three non-resuscitated animals died in 45- and 60-min post-injury and the other animal remained hypotensive (MAP 45 mmHg), tachycardic (HR 125 beats·min^−1^) and with elevated lactate level (lactate 2.2 mmol·dl^−1^) at 3-h post-laceration. Although resuscitation to MAP and HR targets occurred most animals required continuous and often times increasing levels of volume and vasopressor support over time, consistent with the uncontrolled hemorrhage insult.

**Table 1:** Fluid and norepinephrine doses, serum lactate, SvO_2_ and cardiac output across groups

	ReFit 2 lab only	ReFit 2 transport	Not resuscitated
Number of animals	8	4	3
Time to initial sufficiency			
Time min	48.7 ± 18.4	42.3 ± 30.1	
Fluid ml/kg	5.3 ± 8.1	9.8 ± 12.7	0
Total NE microg/kg	0.46 ± 1.0	1.15 ± 1.2	0
Mean NE rate Microg/kg/min	0.06 ± 0.01	0.019 ± 0.013	0
Initial sufficiency to 2 h			Time to death (min)
Fluid ml/kg	2.5 ± 6.6	13.8 ± 10.5	270 +
Total NE microg/kg	0.52 ± 0.83	2.21 ± 0.60	45
Mean NE rate Microg/kg/min	0.013 ± 0.021	0.041 ± 0.022	90

Mean ± standard deviation for fluid and norepinephrine given to time of initial stabilization and from initial stabilization point until the end of 2 h for the entire cohort. For not resuscitated animals listing only time to death or end of experiment. norepinephrine: NE

**Fig. 1:**
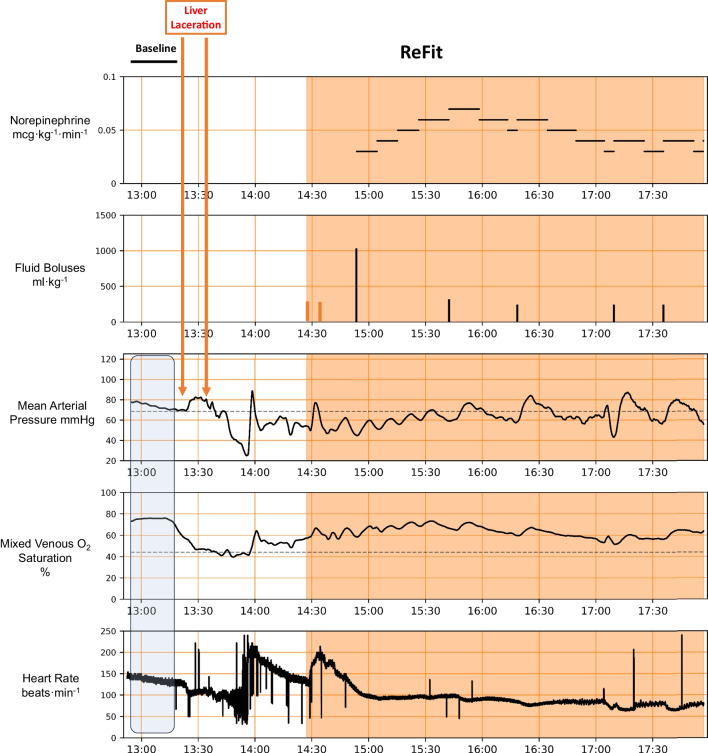
Physiologic trend display of norepinephrine infusion rate, fluid boluses, mean arterial pressure, mixed venous O_2_ saturation, heart rate and periodic serum lactate levels for a complete ReFit experiment from baseline, bleed, delay and resuscitation phases. The initial shaded area reflects the bleed challenge and the second shaded area the resuscitation effects. The red vertical lines in the fluid boluses graph represents infusion of ~ 250 ml whole blood. The one time CaCl_2_ infusion was given immediately after the first blood infusion and is not marked separately

**Fig. 2:**
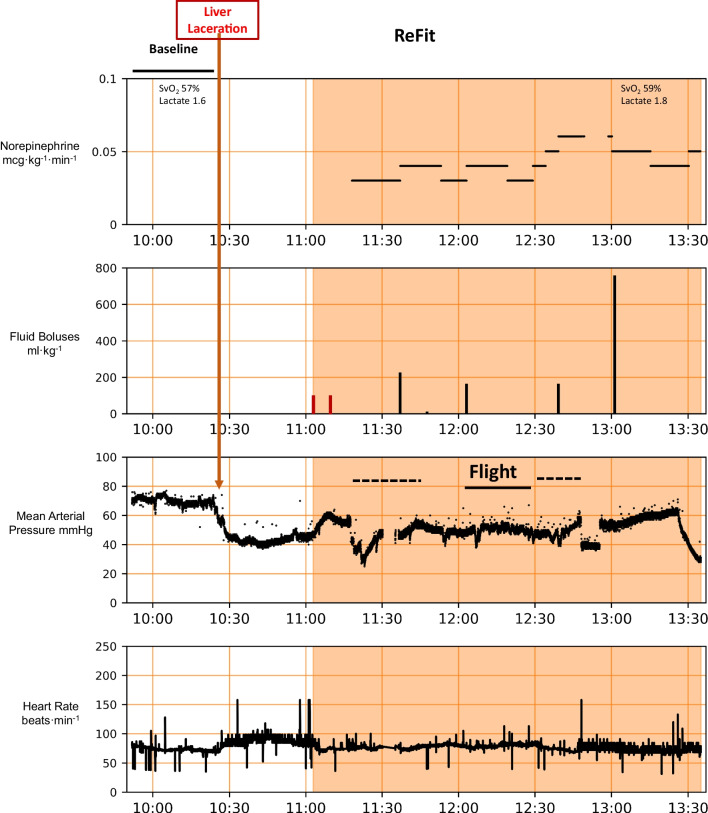
Physiologic trends display similar to Fig. 1, but without mixed venous O_2_ saturation trending for the first animal to be transported to the helipad, flown by rotary wing transport for 24 min covering 39.2 nm and returned to the laboratory. The various phases of the protocol are shown. The time when the ReFit algorithm was active is displaced by the shared area. The time in transit is shown as a horizontal dashed line and during flight as a solid line

**Fig. 3:**
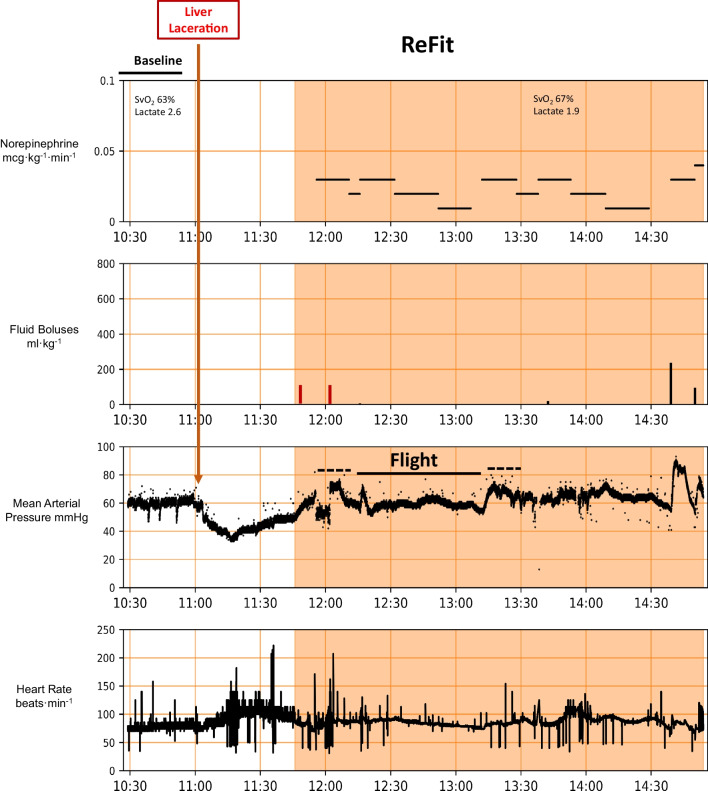
Physiologic trends display similar to Fig. 2 for the second animal to be transported by air only for 36 min covering 66.4 nm

**Fig. 4:**
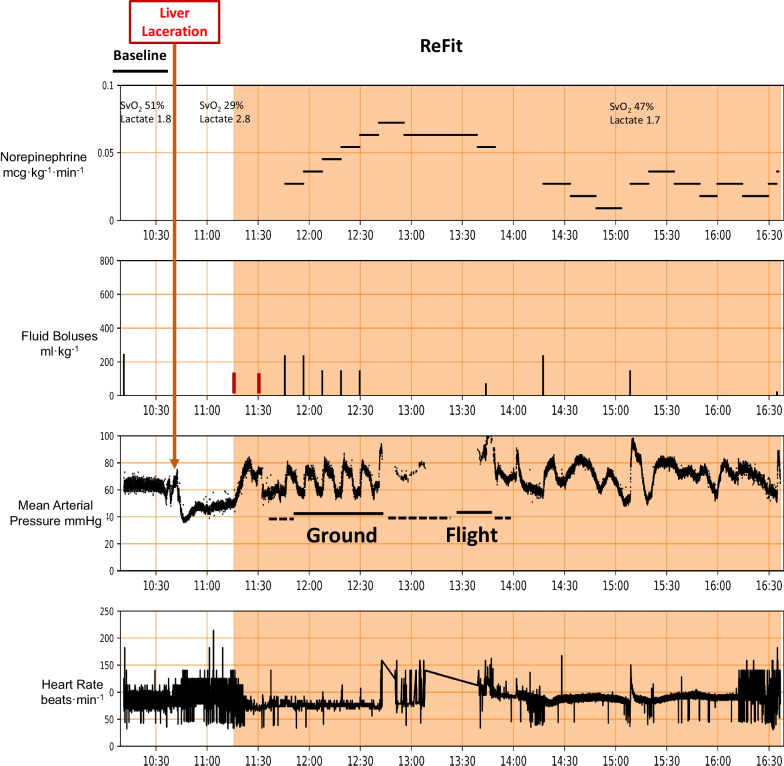
Similar physiologic trends display similar to Figs. 2 and 5, but for the third animal this time transported first by ground to a remote airport covering 9.1 miles in 30 min then flown back to the hospital by rotary wing transport for 12 min covering 9.1 miles. The total time in transit to the ground ambulance is shown as a horizontal dashed line and the time in transport on ground and in the air as two solid horizontal lines

**Fig. 5:**
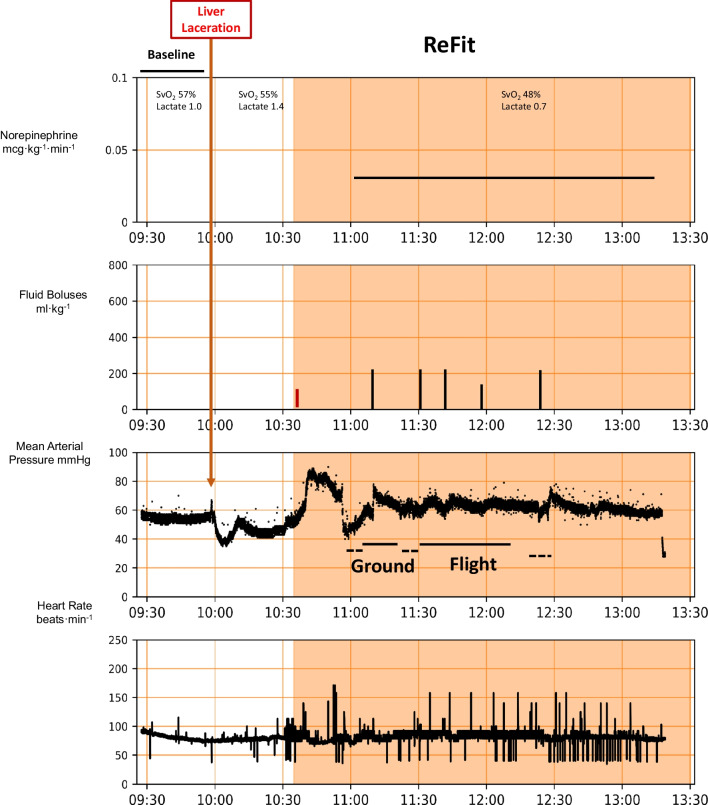
Similar physiologic trend displays as Fig. 4 for the fourth animal to be transported first by ground to a remote airport covering 9.1 miles in 30 min then flown back to the hospital by rotary wing transport for 35 min covering 62.3 miles

No animal in transport had untoward events or required intervention by the safety officer. However, all transported animals had transient decreases in MAP during the physical act of transfer from the surgical table to the stretcher, not requiring any specific intervention other than that delivered by the Refit protocol. In the two ground–air transport animals, we also were able to remotely monitor and control a Neurowave infusion pump for ketamine infusion. An example of animal one during laboratory only resuscitation is shown in Fig. 1, and all four transported animals are shown in Figs. 2, 3, 4, 5, illustrating the effectiveness of the ReFit protocol and showing that the ability to reach stability quickly using the ReFit protocol was independent of transportation efforts. We had two notable events during land to air transport. First, animal 3 (Fig. 4) had the arterial pressure USB cord disrupted from the monitor to the ruggedized computer as it was being moved out of the ambulance. This froze the algorithm orders and required an internal automatic reboot which took 10 min to discover (there are presently no alarms on the Refit device) and an additional 15 min to reboot once the USB plug was repaired and reconnected. This is shown as the blank place in the recording which includes a 15-min observation interval to define physiological state. As can be seen the infusion of norepinephrine remained constant and caused a higher pressure than targeted once the arterial pressure monitoring was renewed. The ReFit algorithm once restarted weaned norepinephrine accordingly. Second, animal 4 responded following the initial 250 ml whole blood infusion followed by 1 mg CaCl_2_ because sufficient for the next 45 min and as per the ReFit algorithm defaulted to giving only lactated Ringer’s solution as resuscitation bolus fluid and no second unit of whole blood. Since we did not monitor the pulmonary artery catheter data during the experiments on the transport assigned animals, we do not report continuous SvO_2_ values in those animals. However, in all 4 animals upon return to the laboratory spot SvO_2_ measures by manual sampling were 72, 65, 74, and 65%, respectively, as shown in Figs. 2, 3, 4, 5. Though not used to inform the protocol, cardiac output and SvO_2_ levels approached baseline values during resuscitation in all the laboratory only animals (Table 2).

**Table 2:** Hemodynamic data at across the protocol

	MAP mmHg	HR beats $\cdot$ min^−1^	SaO_2_%	SvO_2_%	Hbg gm/dl	CO l·min^−1^
ReFit Lab only *N* = 8						
Baseline	68.8 ± 3.7	82.2 ± 17.6	100 ± 0	57 ± 6	10.6 ± 1.2	2.75 ± 1.31
Pre-resuscitation	48.6 ± 4.9	102.4 ± 21.6	100 ± 0	43 ± 10	9.1 ± 1.6	1.62 ± 0.93
At Initial stabilization	75.5 ± 9.2	77.0 ± 11.1	100 ± 0	64 ± 5	8.3 ± 1.6	2.75 ± 0.58
2 h post-initial stabilization	68.1 ± 6.0	74.9 ± 10.2	100 ± 0	59 ± 11		2.83 ± 139
ReFit 2 Lab and transport *N* = 4						
Baseline	62.4 ± 3.7	87..8 ± 3.6	97 ± 2		8.6 ± 1.5	
Pre-resuscitation	48.9 ± 2.0	86.6 ± 3.4	97 ± 2		8.7 ± 1.3	
At initial stabilization	70.4 ± 12.3	80.8 ± 3.7	96 ± 2		9.3 ± 0.9	
2 h post-initial stabilization	60.6 ± 5.4	78.4 ± 2.8	94 ± 2			

Mean ± standard deviation of mean arterial pressure (MAP), heart rate (HR), mixed venous O_2_ saturation (SvO_2_) and cardiac output (CO) for the three experimental groups over the same time points in the protocol. Note SvO_2_ and CO were not continuously measures in the 4 animals study for transport

## Discussion

Our data demonstrate that our PCLC ReFit algorithm using a minimally invasive monitoring approach in a porcine model of lethal uncontrolled hemorrhagic shock resulted in rapid and sustained stabilization for up to 3 h when conducted in both a laboratory or remotely during air or ground to air transport. To our knowledge, this is the first time a remote autonomous resuscitation and stabilization system applied during transport of a severely injured animal is reported to be successful. The potential this brings to the practice of care in remote field setting without access to advanced medical expertise is significant.

Furthermore, the ReFit algorithm functions as a precision medicine tool. It uses dynamic parameters to guide fluid administration and wean vasopressors and also infuses fluids proportional to the degree of volume responsiveness. Thus, volume boluses are individualized based on the degree of volume responsiveness and not given if the subject is not perceived to be volume responsive, even if in shock. Similarly, weaning of norepinephrine is done only if MAP remains stable and vasomotor tone, estimated by Ea_dyn_ is > 1, thereby minimizing the risk of reactive hypotension. This approach is not only precise, by giving only treatments that are needed, but personalized by giving a dose of treatment proportional to the subject’s volume responsiveness or intrinsic vasomotor levels. This ReFit approach should minimize fluid overload, iatrogenic hypotension, and conserve resources. The desired resuscitation MAP and HR targets can be adjusted, as ongoing bleeding is often resuscitated to lower MAP targets (i.e., hypotensive resuscitation) or when resuscitative products are limited. Once bleeding is controlled, in the setting of traumatic brain injury or additional resources are available, higher MAP targets may be needed for end organ perfusion with comorbid disease such as pre-existing hypertension. The ReFit algorithm can be set to target any defined MAP.

The ReFit algorithm has several advantages over fixed protocolized approaches. First, it does not require advanced care expertise to initiate and run. What is needed is an indwelling arterial catheter, the ReFit infusion pumps, monitoring array module and resuscitation fluids. Second, it is not a “black box” algorithm, but individualizes resuscitative care since every therapeutic intervention is linked to a specific expected physiologic response of the subject being treated. It is sensor specific but device agnostic, any monitoring device that accurately reports MAP, SVV and PPV within a 20–30 s moving window can be used to drive the algorithm and many available monitoring devices fit this requirement [10]. In addition to improving vital signs, the algorithm supports end organ perfusion, as evinced by the improvement in cardiac output and SvO_2_, demonstrating the robustness of this approach. Future applications include fully autonomous unmanned vehicle-based transport.

Our approach has several limitations. First, we demonstrated this in an anesthetized porcine model of uncontrolled hemorrhagic shock. To the extent that similar effectiveness would be seen in human trauma patients with a myriad of insults needs to be validated. Second, it requires the subject to be on controlled mechanical ventilation. Dynamic measures such as PPV and SVV deteriorate in their predictive power during spontaneous ventilation [11]. However, Ea_dyn_ remains accurate at all times [12]. We are actively searching for signatures of volume responsiveness that can be easily applied autonomously during spontaneous breathing. Candidate diagnostics such as a passive leg raising or mini-fluid challenge presently do not translate well to austere environments. Third, the ReFit algorithm treats only shock due to hypovolemia and vasoplegia, not due to obstruction or primary cardiac events. Thus, patient selection will need to be done carefully and other treatments for tension pneumothorax and cardiac injury, for example, will need to be addressed separately. Fourth, measures of PPV and SVV are essential to drive this algorithm. FDA-approved non-invasive devices that report these parameters using finger cuff exist. However, in profound hypotensive shock, peripheral perfusion is often lost as is pulse oximetry measures of SpO_2_. If field responses presume profound hypotension requires immediate fluid boluses as are also applied to a modified ReFit algorithm, then those devices will resume reporting MAP, pulse rate, PPV and SVV once MAP exceeds 50 mmHg. However, it is not clear if such levels of profound hypotension can be effectively managed for our algorithm beyond the initial fluid boluses. In practice, all animals increased their MAP to > 50 mmHg after the first blood and CaCl_2_ bolus. Finally, as illustrated in the third transported pig, the ReFit algorithm has no disconnect alarms. This was easily addressed in a recent revision of the ReFit software and also secured with more permanent connection appliances. Also, as a default mode, if no physiologic input is sensed the ReFit algorithm defaults to a fixed mode of constant norepinephrine at the dose it was already on and no new fluid boluses. Clearly, if any of the components of the PCLC system (input data, infusion pumps or resuscitation fluids) are removed the system cannot operate. Still, if unmanned care during transport of critically ill trauma patients is needed, the ReFit algorithm results in better stabilization that that seen by no treatment at all.

In conclusion, our data demonstrated that ReFit, a physiology-based, personalized, closed-loop resuscitation system can effectively stabilize an acutely anesthetized animal subjected to lethal uncontrolled hemorrhagic shock in both laboratory conditions and during real-life ground and air transportation without human intervention. Our results also suggest that this system can be monitored and operated remotely, adding an important feature of ‘human-in-the-loop’ capacity that potentially could be more adaptive if monitoring inputs are limited or pathophysiologic processes are more complex. Overall, this first report of successful application of an autonomous resuscitation during transport opens promising possibilities to extend the concept of the ‘golden hour’ to a realistic three hours, and sustain organ perfusion until definitive treatment is achieved. Finally, it raises the prospect of a system that can expand the capability of delivery of personalized care in situations where resources may be constrained.

## Take home message

We demonstrate that a physiologically based closed-loop diagnosis and resuscitation algorithm can effectively resuscitation lethal uncontrolled hemorrhage in an acute anesthetized and mechanically ventilated porcine model and do so even during real-life air and ground transport. The implications of these findings and this approach to autonomously support advanced resuscitation in remote and care-limited environments, potentially prolonging survival of patients prior to definitive care is significant.

## Tweet

Our physiologically based closed-loop system can resuscitate hemorrhagic shock animals during lab care and during air or ground transport.

### Supplementary Information

**Additional file 1**: **Figure S1.** ReFit Main Algorithm. **Figure S2. **Amount of fluid bolus a function of the degree of hypovolemia as defined by level of volume responsiveness (PPV, SVV). **Figure S3.** Dose of Norepinephrine, a function of MAP for increase Decreasing Norepinephrine, a function of MAP and Eadyn (PPV/ SVV). **Figure S4.** Photograph of the Neurowave Infusion Pump Manifold as Used in the 4 Animals During Ground and Air Transported. **Figure S5. **Photograph of the ReFit Graphic Display from the Ruggedized Computer During Air Transport. **Figure S6.** Photograph of the Animal, ReFit Surgical and Transport Teams as they arrived at the Rooftop Helipad for Air Transport. **Figure S7. **Photograph of an Animal in Hemorrhagic Shock Being Transferred into the Helicopter. **Figure S8.** Photograph of a Hemorrhagic Shock Animal with ReFit Monitoring and Treatment Configuration within the Helicopter During Air Transport. **Figure S9.** Flight paths for the four animals Transported by Air (top) and by Ground from Hospital to Remote Airfield (bottom).

## Data Availability

The ReFit algorithm is described in the supplemental material. The specific animal data are also displayed but not applicable to duplication of this study by other investigators.

## References

[1] Army US(2020) Tactical Combat Casualty Care Handbook. LULU PRESS Incorporated.

[2] Poropatich RK, Pinsky MR (2020) Robotics enabled autonomous and closed loop trauma care in a rucksack - TRACIR. Healthcare 396. Transform (AI, Automation & Robot) 10.1089/heat.2019.0007

[3] https://www.economist.com/international/2023/08/04/what-ukraines-bloody-battlefield-is-teaching-medics

[4] Pinsky MR, Payen D (2005). Functional hemodynamic monitoring. Crit Care.

[5] Haugaa H (2015). Effects of inhalation of low dose nitrite or carbon monoxide on post-reperfusion mitochondrial function and tissue injury in hemorrhagic shock swine. Crit Care.

[6] Pinsky MR, Gomez H, Leonard J, Wertz A, Dubrawski A, Poropatich R (2024) Evaluation of a physiological driven closed loop resuscitation algorithm. Crit Care Med. (in press) 10.1097/CCM.0000000000006297 39436216

[7] https://arriveguidelines.org/

[8] Holcomb JB (1999). Effect of dry fibrin sealant dressings versus gauze packing on blood loss in grade V liver injuries in resuscitated swine. J Trauma.

[9] Clarke EE (2022). Trends in prehospital blood, crystalloid, and colloid administration in accordance with changes in tactical combat casualty care guidelines. Mil Med.

[10] Hadian M, Kim H, Severyn DA, Pinsky MR (2010). Cross-comparison of cardiac output trending accuracy of LiDCO, PiCCO FloTrac and pulmonary artery catheters. Crit Care.

[11] Monnet X, Malbrain MLNG, Pinsky MR (2023). The prediction of fluid responsiveness. Intensive Care Med.

[12] Monge Garcia M (2014). Dynamic arterial elastance as a predictor of arterial pressure response to fluid administration: a validation study. Crit Care.

