# Supplementary material for: Autonomous precision resuscitation during ground and air transport of an animal hemorrhagic shock model
Source: Intensive Care Med Exp. 2024 May 24;12:44. doi: 10.1186/s40635-024-00628-5 (PMC11116353; doi:10.1186/s40635-024-00628-5)
Supplement: Supplementary file 1 — Additional file 1: Figure S1. ReFit Main Algorithm. Figure S2. Amount of fluid bolus a function of the degree of hypovolemia as defined by level of volume responsiveness (PPV, SVV). Figure S3. Dose of Norepinephrine, a function of MAP for increase Decreasing Norepinephrine, a function of MAP and Eadyn (PPV/ SVV). Figure S4. Photograph of the Neurowave Infusion Pump Manifold as Used in the 4 Animals During Ground and Air Transported. Figure S5. Photograph of the ReFit Graphic Display from the Ruggedized Computer During Air Transport. Figure S6. Photograph of the Animal, ReFit Surgical and Transport Teams as they arrived at the Rooftop Helipad for Air Transport. Figure S7. Photograph of an Animal in Hemorrhagic Shock Being Transferred into the Helicopter. Figure S8. Photograph of a Hemorrhagic Shock Animal with ReFit Monitoring and Treatment Configuration within the Helicopter During Air Transport. Figure S9. Flight paths for the four animals Transported by Air (top) and by Ground from Hospital to Remote Airfield (bottom). [file 40635_2024_628_MOESM1_ESM.pdf]

Figure S1. ReFit Main Algorithm

Main decision tree

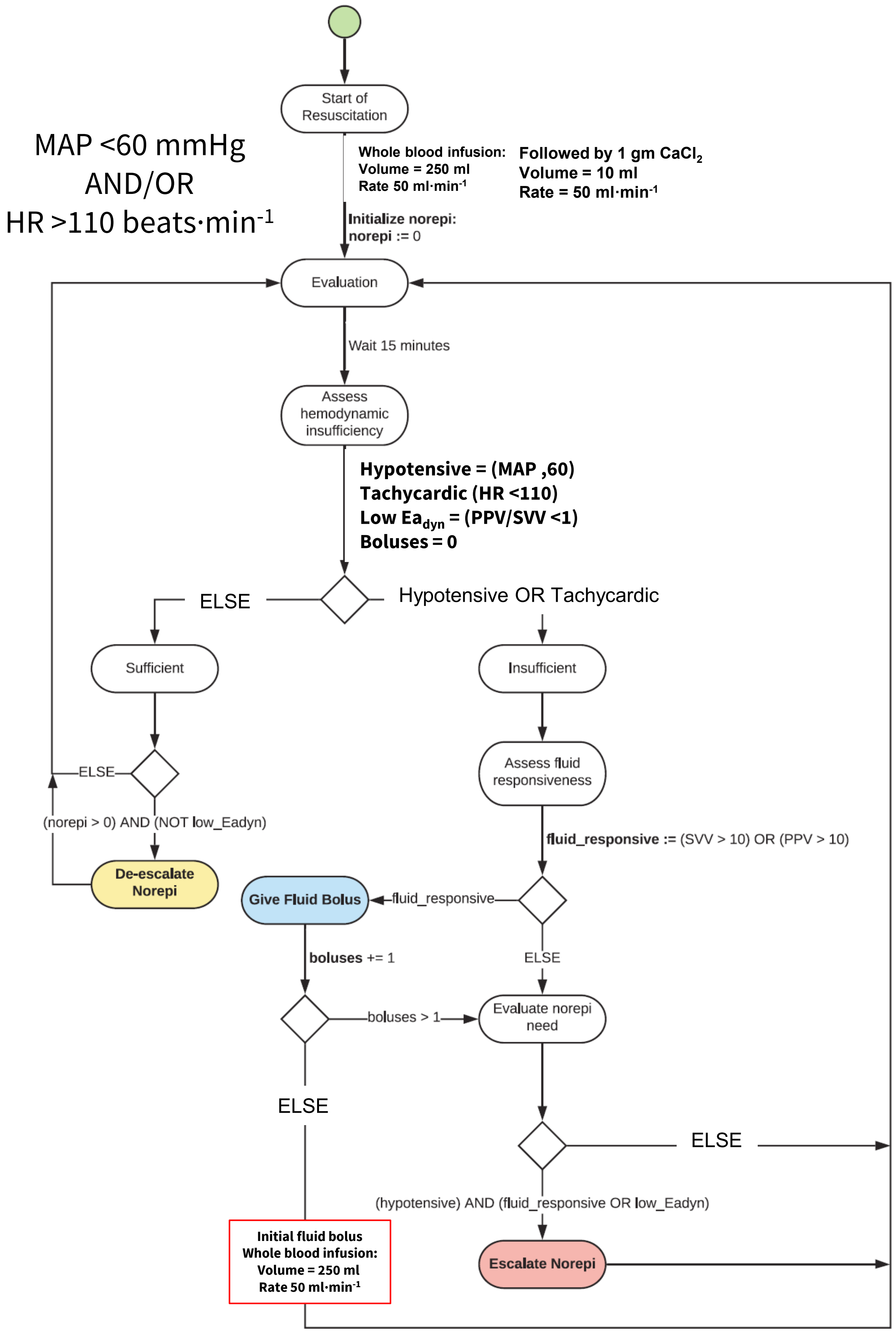

Created by Anthony Wertz, Jarod Wang and Jim Leonard, CMU
Using Michael Pinsky's ReFit Protocol

Figure S2. Amount of fluid bolus a function of the degree of hypovolemia as defined by level of volume responsiveness (PPV, SVV)

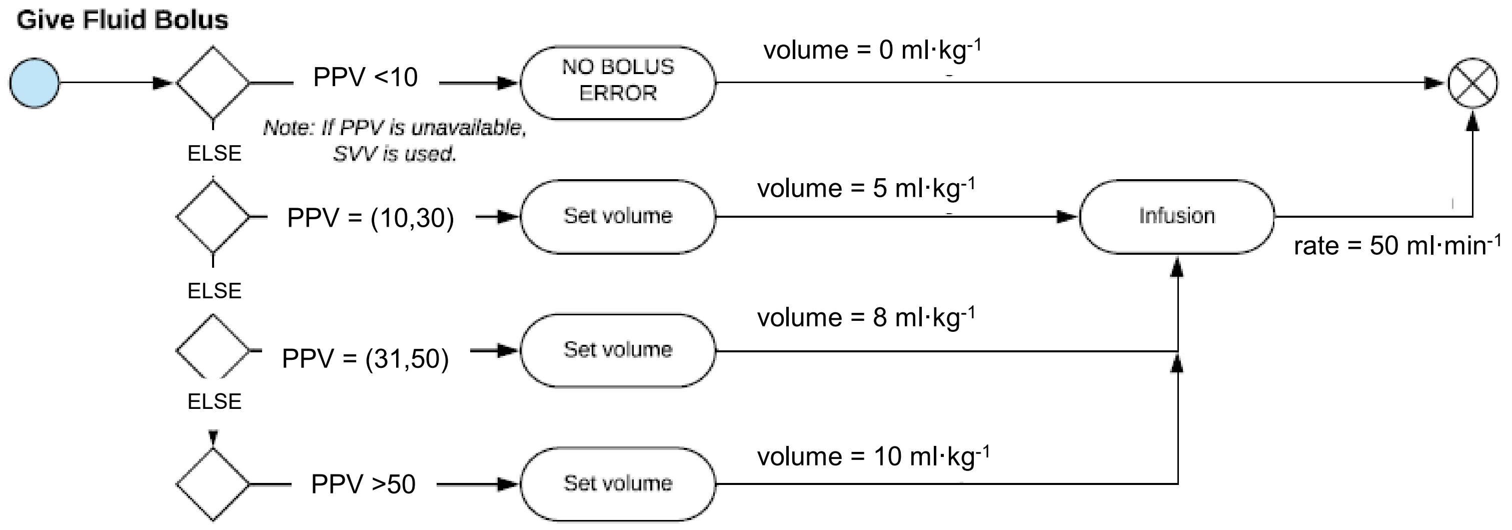

Figure S3. Dose of Norepinephrine, a function of MAP for increase Decreasing Norepinephrine, a function of MAP and Ea_{dyn} (PPV/ SVV)

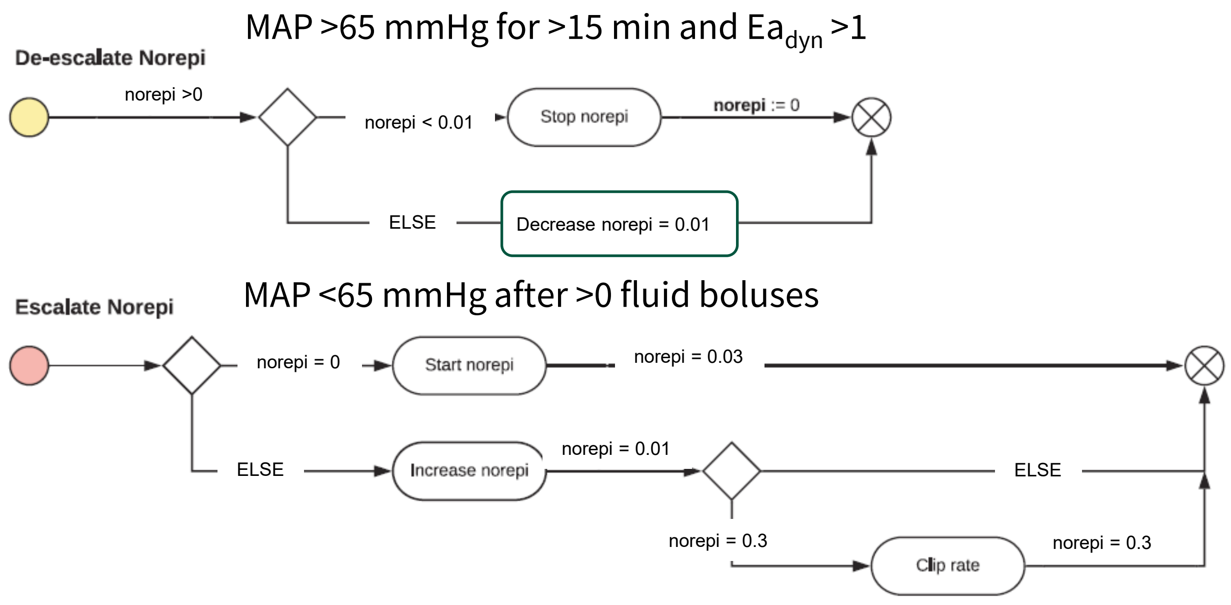

Figure S4. Photograph of the Neurowave Infusion Pump Manifold as Used in the 4 Animals During Ground and Air Transported

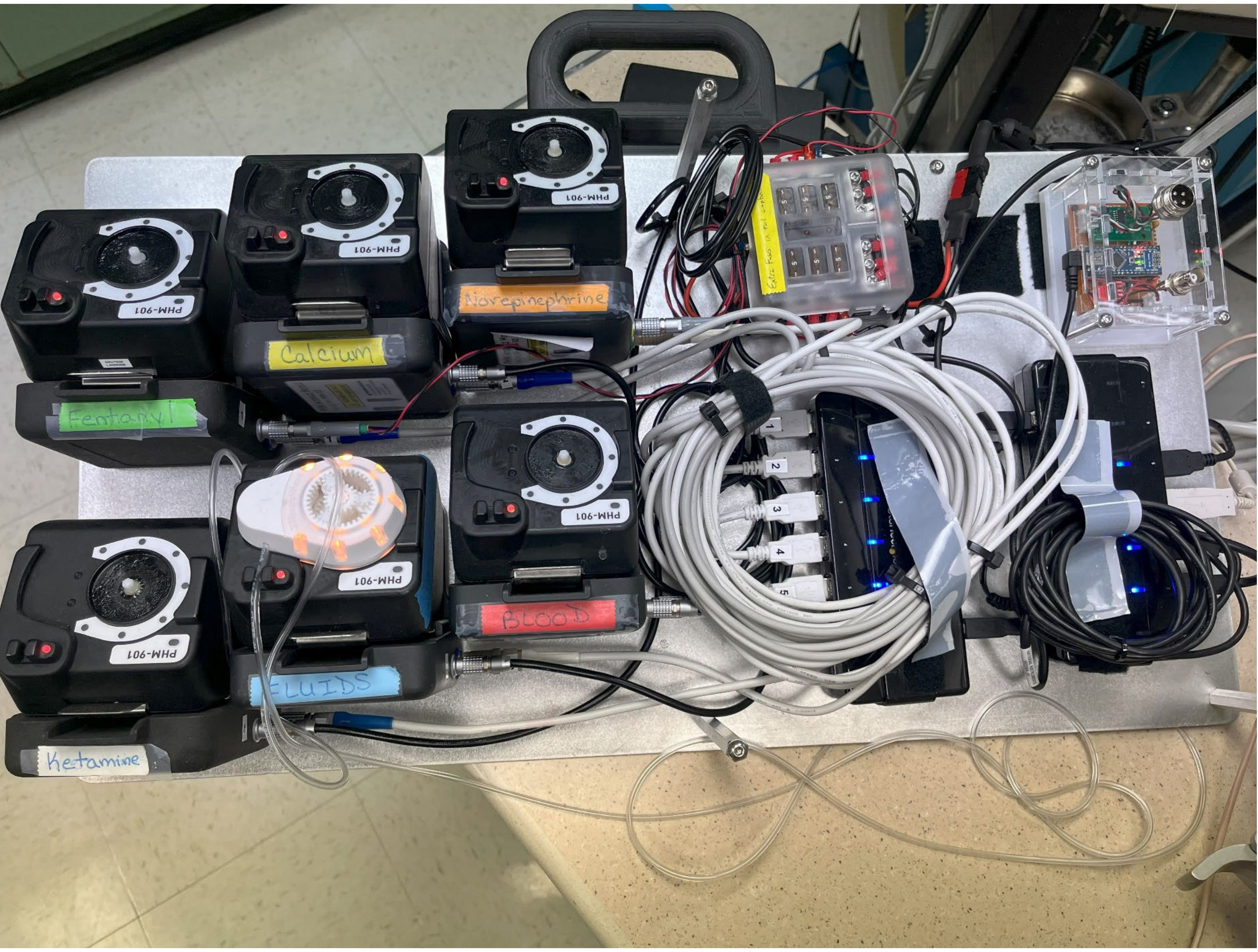

The six Neurowave pumps are labeled in color: fentanyl (green), ketamine (white), calcium (yellow), fluids (blue), norepinephrine (orange) and blood (red). The manifold connected to a ruggedized computer whose input was from the arterial pressure wave form from an indwelling arterial catheter and electrocardiographic leads that were processed through the LiDCO*plus*™ minimally-invasive monitor.

Figure S5. Photograph of the ReFit Graphic Display from the Ruggedized Computer During Air Transport

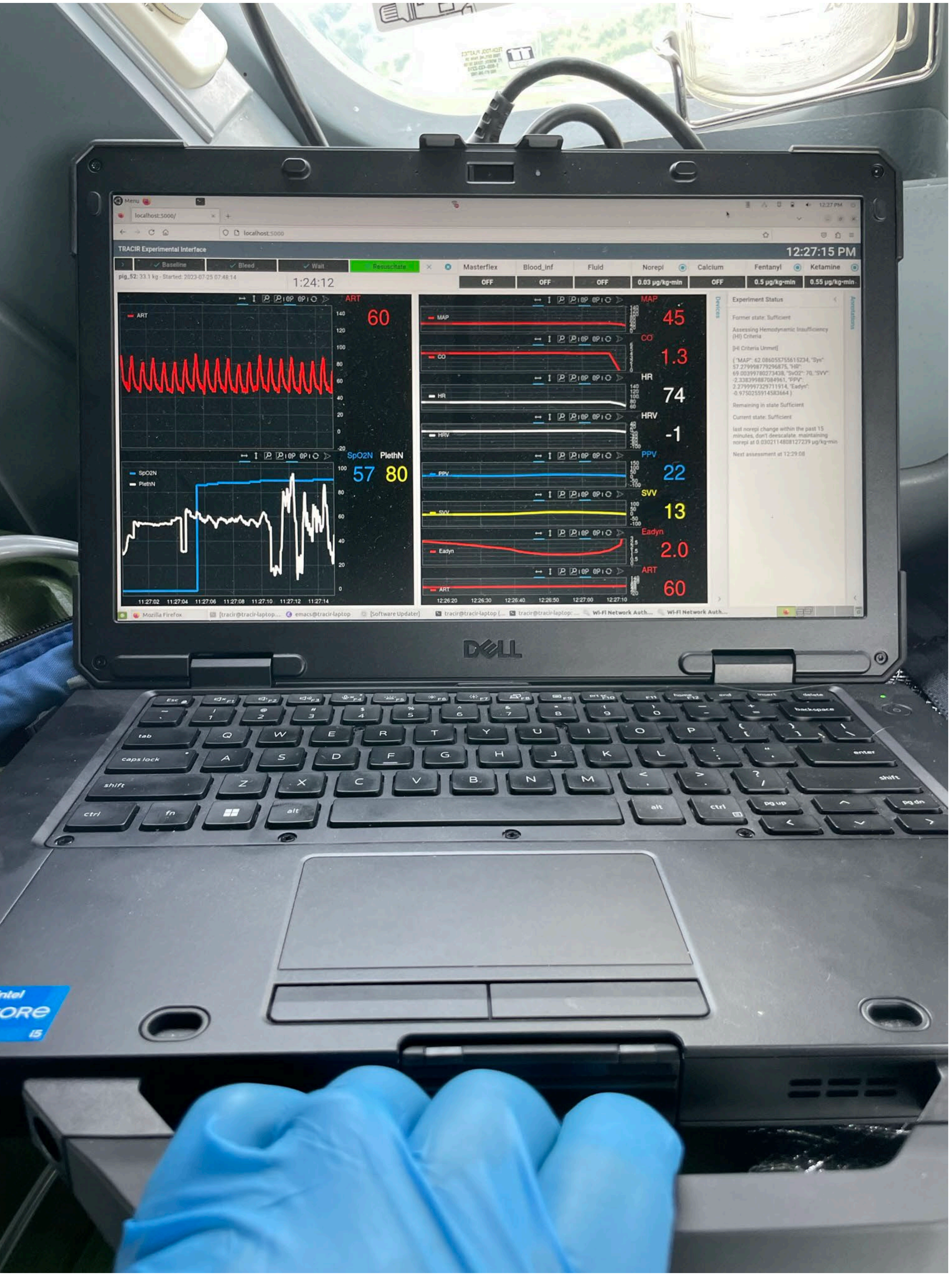

The left panel of the display is the real-time arterial pressure and pulse oximeter waveform data along with their respective 20 second moving window mean values. The middle panel displays the primary hemodynamic variables and parameters used to drive the ReFit algorithm. The right panel is the active decision tree-based commands and logic tree reasoning for the present resuscitation cycle commands.

Figure S6. Photograph of the Animal, ReFit Surgical and Transport Teams as they arrived at the Rooftop Helipad for Air Transport

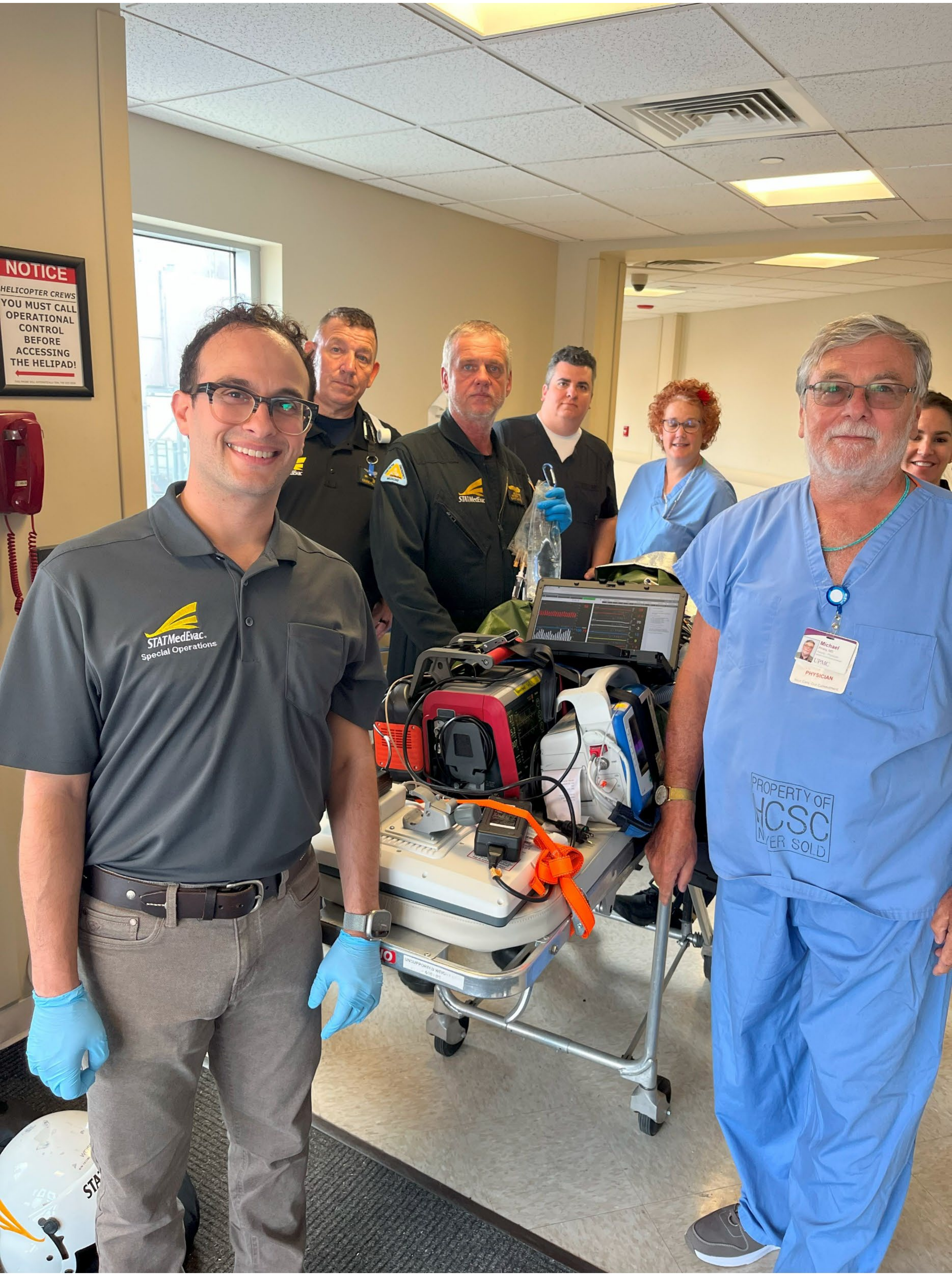

The STAT MedEvac transport team (left side) and the surgical team (in surgical scrubs) with the animal and ReFit monitorng/treatment apparatus on the stretcher upon reaching the helipad for the first air transport study.

Figure S7. Photograph of an Animal in Hemorrhagic Shock Being Transferred into the Helicopter

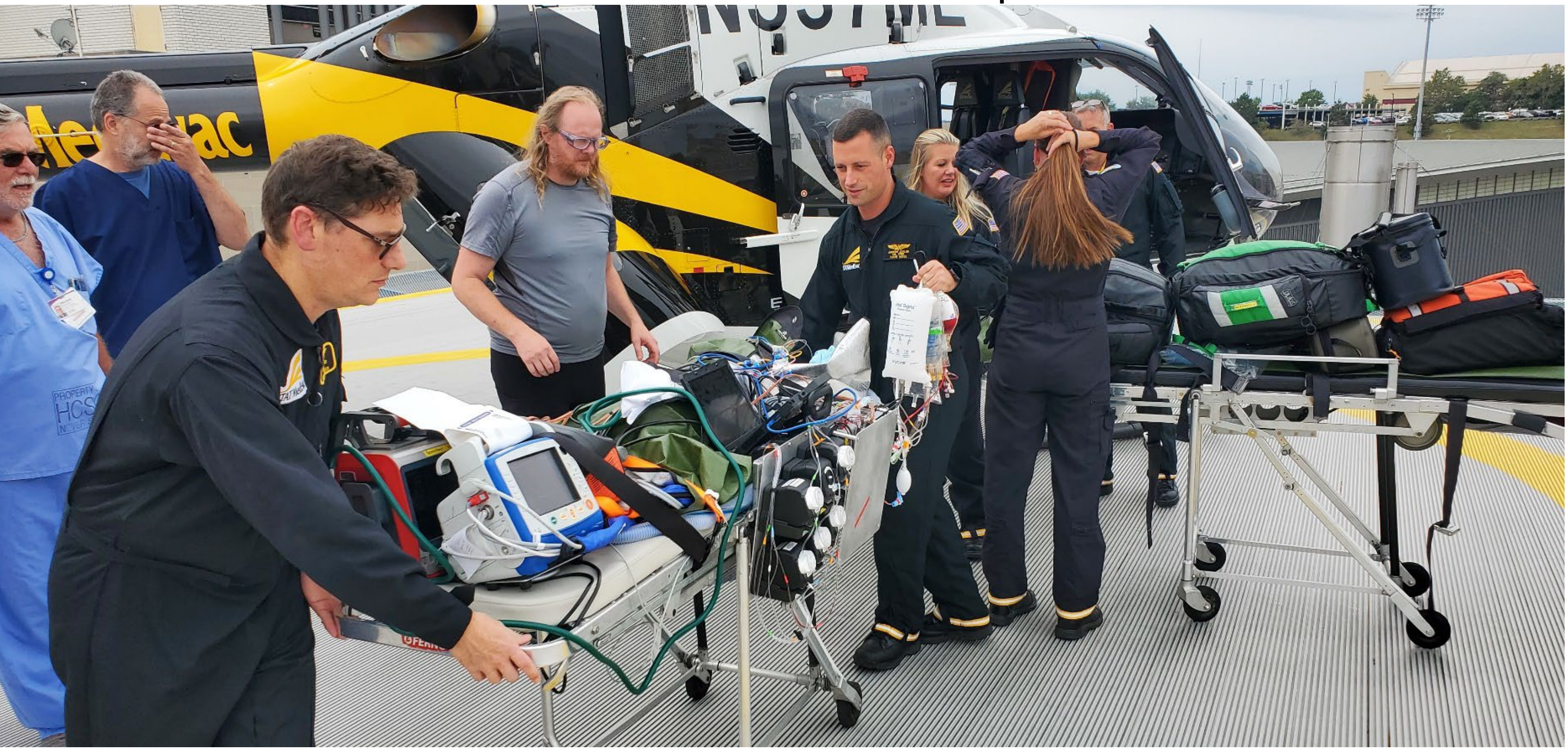

Figure S8. Photograph of a Hemorrhagic Shock Animal with ReFit Monitoring and Treatment Configuration within the Helicopter During Air Transport

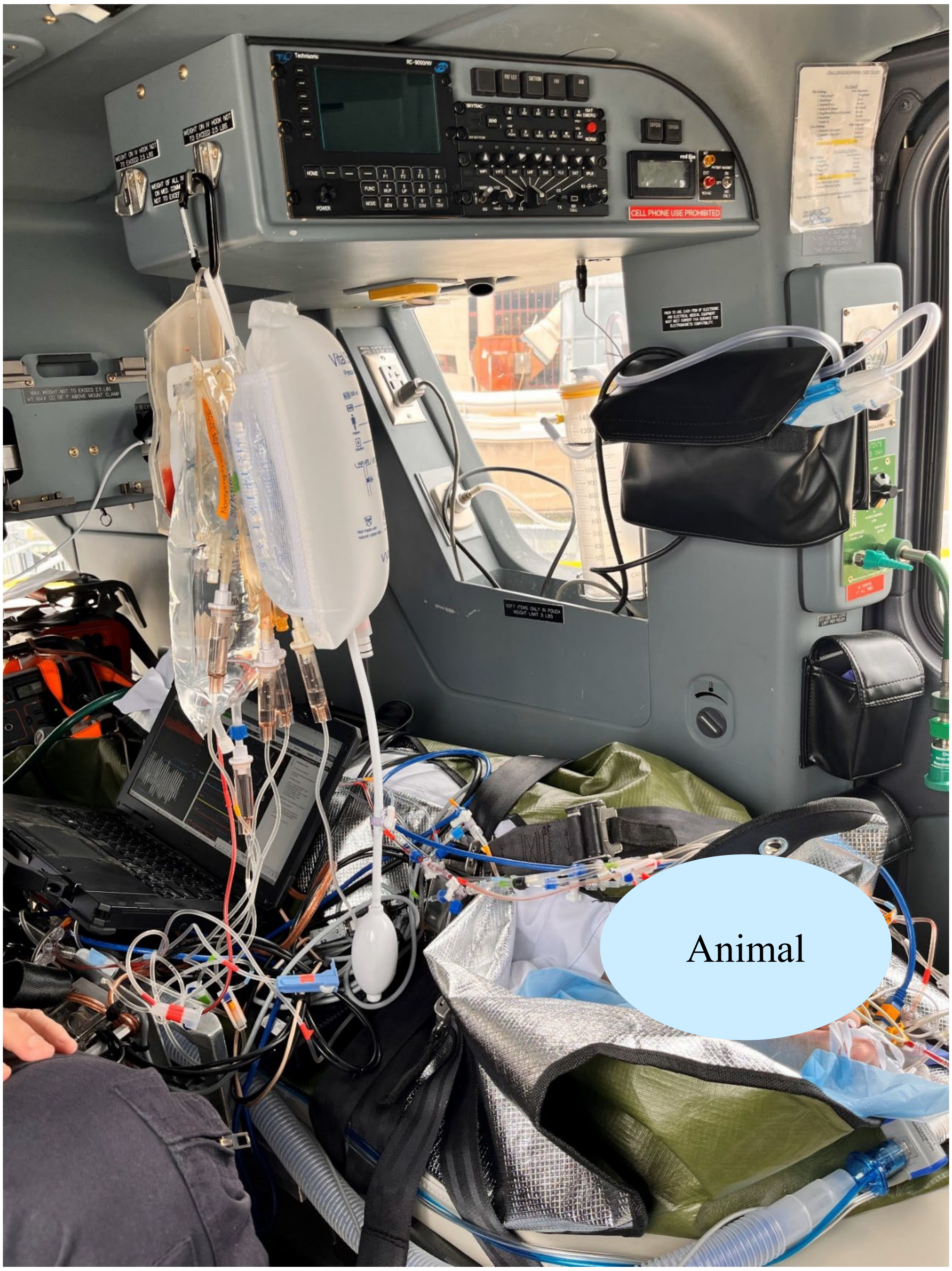

Figure S9. Flight paths for the four animals Transported by Air (top) and by Ground from Hospital to Remote Airfield (bottom)

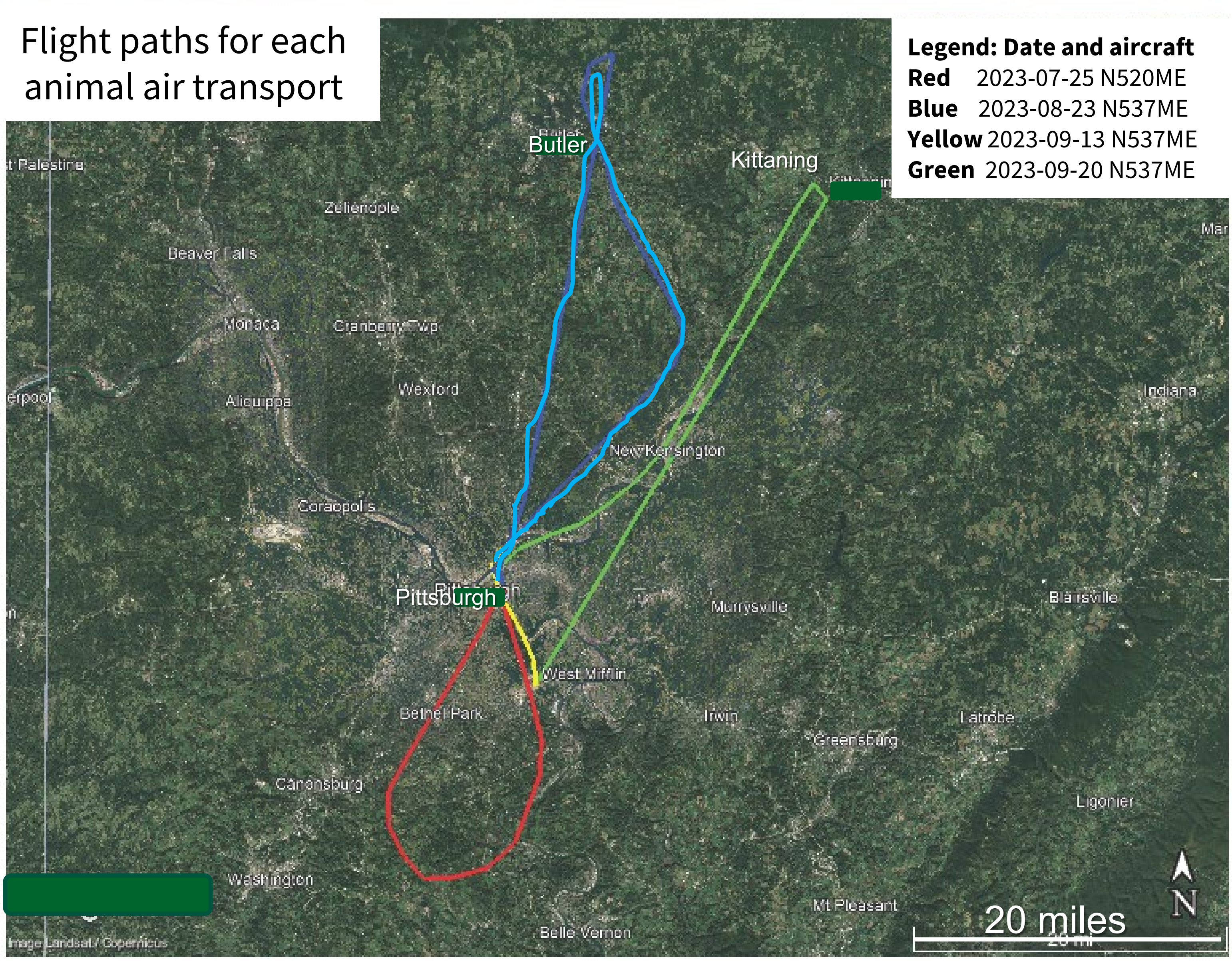

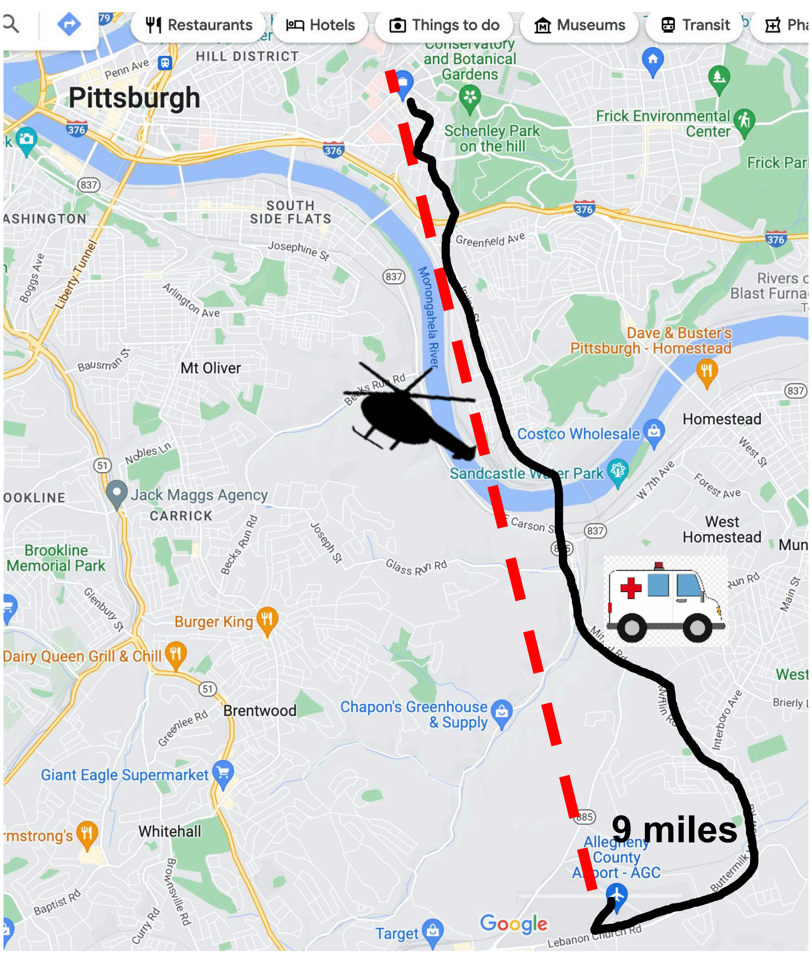

The two animals shown in **red** and **blue** were flown from the UPMC helipad and returned to UPMC helipad. The two animals shown in **yellow** and **green** were driven by ambulance from UPMC to a remote airport (see bottom map) and then flown back to UPMC helipad. The second air-only (**red**) and ground-to-air (**green**) flights were extended to simulate longer flights needed to transport a traumatized patient to the hospital from a remote site.
